# Omics approaches in *Allium* research: Progress and way ahead

**DOI:** 10.7717/peerj.9824

**Published:** 2020-09-09

**Authors:** Kiran Khandagale, Ram Krishna, Praveen Roylawar, Avinash B. Ade, Ashwini Benke, Bharat Shinde, Major Singh, Suresh J. Gawande, Ashutosh Rai

**Affiliations:** 1 Department of Botany, Savitribai Phule Pune University, Pune, Maharashtra, India; 2 ICAR-Directorate of Onion and Garlic Research, Rajgurunagar, India; 3 Department of Botany, S. N. Arts, D. J. M. Commerce and B. N. S. Science College, Sangamner, India; 4 Vidya Pratishthans’s Arts Science and commerce college, Baramati, India; 5 Crop Improvement, ICAR-Indian Institute of Vegetable Research, Varanasi, India

**Keywords:** Allium, Genomics, Transcriptomics, Proteomics, Metabolomics, Metagenomics, Microrna

## Abstract

**Background:**

The genus *Allium* (Family: Amaryllidaceae) is an economically important group of crops cultivated worldwide for their use as a vegetable and spices. Alliums are also well known for their nutraceutical properties. Among alliums, onion, garlic, leek, and chives cultivated worldwide. Despite their substantial economic and medicinal importance, the genome sequence of any of the *Allium* is not available, probably due to their large genome sizes. Recently evolved omics technologies are highly efficient and robust in elucidating molecular mechanisms of several complex life processes in plants. Omics technologies, such as genomics, transcriptomics, proteomics, metabolomics, metagenomics, etc. have the potential to open new avenues in research and improvement of allium crops where genome sequence information is limited. A significant amount of data has been generated using these technologies for various *Allium* species; it will help in understanding the key traits in *Allium* crops such as flowering, bulb development, flavonoid biosynthesis, male sterility and stress tolerance at molecular and metabolite level. This information will ultimately assist us in speeding up the breeding in *Allium* crops.

**Method:**

In the present review, major omics approaches, and their progress, as well as potential applications in Allium crops, could be discussed in detail.

**Results:**

Here, we have discussed the recent progress made in *Allium* research using omics technologies such as genomics, transcriptomics, micro RNAs, proteomics, metabolomics, and metagenomics. These omics interventions have been used in alliums for marker discovery, the study of the biotic and abiotic stress response, male sterility, organ development, flavonoid and bulb color, micro RNA discovery, and microbiome associated with *Allium* crops. Further, we also emphasized the integrated use of these omics platforms for a better understanding of the complex molecular mechanisms to speed up the breeding programs for better cultivars.

**Conclusion:**

All the information and literature provided in the present review throws light on the progress and potential of omics platforms in the research of *Allium* crops. We also mentioned a few research areas in *Allium* crops that need to be explored using omics technologies to get more insight. Overall, alliums are an under-studied group of plants, and thus, there is tremendous scope and need for research in *Allium* species.

## Introduction

*Allium* crops are cultivated globally due to their importance as a vegetable, condiment, and spice with medicinal properties. The genus *Allium* belongs to the family Amaryllidaceae of the order Asparagales. Telomerase sequence variation is responsible for the divergence of *Allium* from other genera belonging to Asparagales. The genus *Allium* comprises more than 920 species (Seregin, Anačkov & Friesen, 2015), and among them, onion, garlic, bunching onion, leek, and shallots are widely cultivated because of their economic importance. The total production value of *Allium* crops in 2014 was US$61348 million (http://www.fao.org/faostat/en/#data/QC), of which dry onion and garlic contributed a major share of 70% and 25%, respectively. China, India, and the United States of America are among the major *Allium* producing countries.

The biannual life cycle, high cross-pollination, and inbreeding depression in onion and vegetative propagation, and lack of flowering, especially in garlic, are the main barriers to the conventional breeding of these major *Allium* crops. Alliums are known to possess one of the largest genomes (10–30 pg/1C) among vegetable crops *(Ricroch et al., 2005)*. Thus, sequencing and assembling of these genomes become difficult, leading to limited availability of markers (Chinnappareddy et al., 2013; Khosa et al., 2016a; Khosa et al., 2016b). Therefore, mapping and genomics-assisted breeding in *Allium* crops are lagging *(Shigyo, Khar & Abdelrahman, 2018)* compared with other crops, such as rice, wheat, and tomato. Presently, we are witnessing tremendous developments and data generation from various omics experiments such as genomics, transcriptomics, proteomics, metabolomics, and metagenomics. These approaches have the potential to increase the speed and accuracy of analysis ofthe complex molecular process, ultimately leading to the development of new strategies in breeding programs for *Allium* improvement.

**Figure 1:**
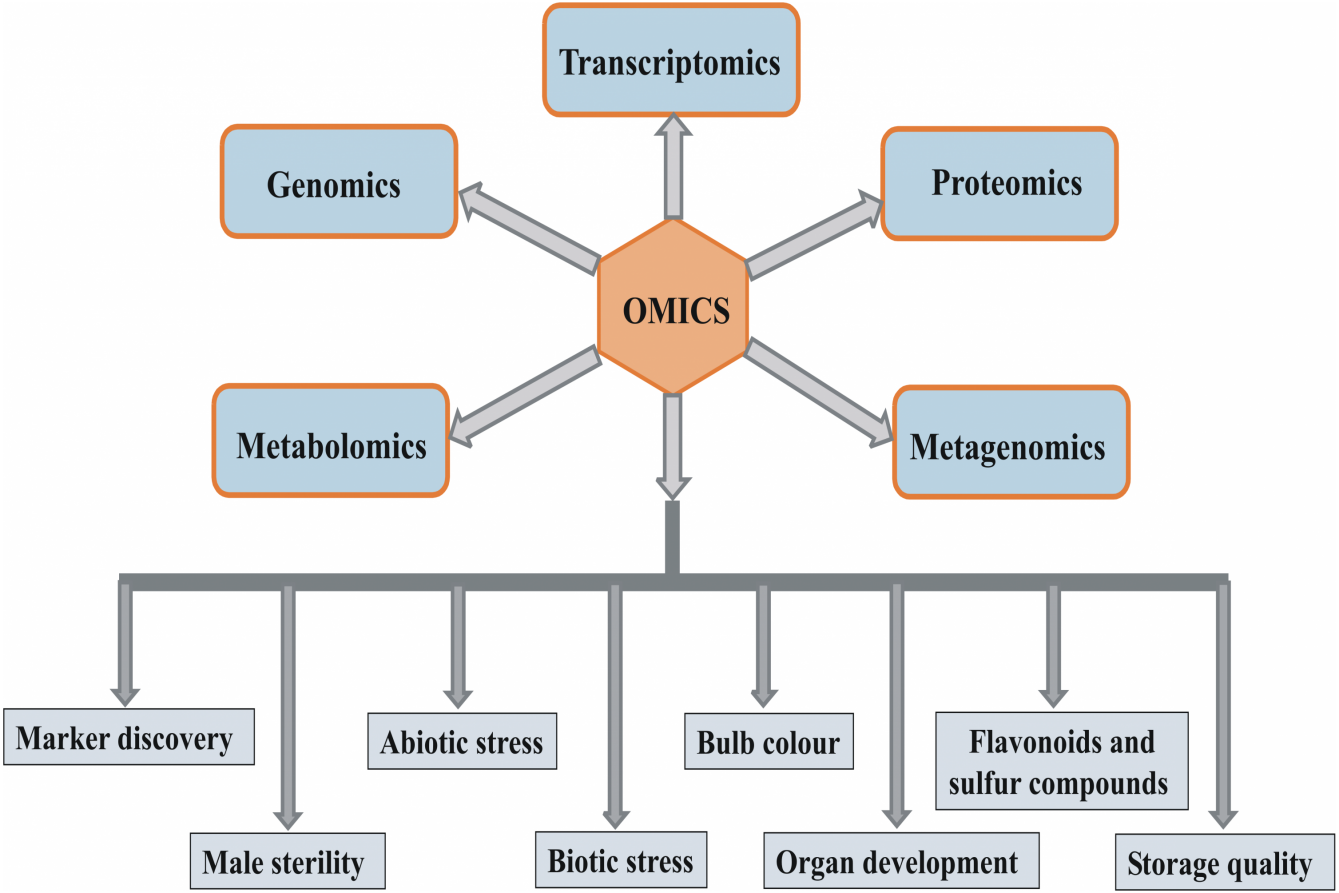
Various omics approaches and their potential application in study of different areas of *Allium* crops improvement.

Next-generation sequencing (NGS) has recently been used for transcriptome analyses of hundreds of model as well as non-model plant species *(Martin et al., 2013)*. RNA sequencing (*RNA-seq)* is a rapid and inexpensive technique that is independent of genome complexity, and thus, NGS has emerged as a method of choice for expression analyses in crops where genome sequence information is unavailable. In alliums*,* RNA-seq was used for studying telomeres, molecular mechanism underlying male sterility, flowering, abiotic stress, and bulb color, and for marker discovery *(Duangjit et al., 2013; Abdelrahman et al., 2015; Kamenetsky et al., 2015; Fajkus et al., 2016; Baek, Kim & Kim, 2017; Yuan et al., 2018)*. The mRNA expression profile differs from the pattern of protein levels, and the correlation between them is often reported in the range of 0.3–0.5 *(Voelckel, Gruenheit & Lockhart, 2017)*. It may be due to protein degradation, post-transcriptional and post-translational regulations *(Voelckel et al., 2010)*. Protein levels are often closely associated with the trait compared to the transcript profile, and therefore, proteomics can be more reliable for studying plant development and responses to stress. Proteomics is an excellent research tool when genome sequence information is limited. A few proteomic studies have been conducted in *Allium* crops to determine abiotic stress tolerance *(Chen et al., 2013; Qin et al., 2016)* and garlic fertility *(Shemesh-Mayer et al., 2015)*. Nowadays, metabolomics is used to gain insights into metabolites involved in specific cellular and developmental processes and stress responses in plants. Because of its rapid and accurate analyses, metabolomics can be used for selecting superior genotypes based on the quantity of metabolites of interest in the breeding program. Metabolomics has a vast potential for the characterization of flavonoids and sulfur (S) compounds in *Allium* crops. Garlic and onion metabolomes were studied for antimicrobial compounds *(Farag et al., 2017)*, authentication of genotypes *(Hrbek et al., 2018)*, and S-containing compounds *(Nakabayashi & Saito, 2017)*. Thus, overall, omics approaches have a massive potential in *Allium* research (Fig. 1)*.* Because *Allium* crops are yet unexplored in terms of omics compared with other crops, omics approaches can assist improvement in these crops.

## Survey Methodology

An extensive literature survey for published research papers in the area of omics approaches in *Allium* crops was conducted using different databases (e.g., Pubmed, Google Scholar, Science Direct, and Scopus). To collect all relevant information, we built our literature search by using the following keyword combinations: genomics and alliums, transcriptomics and alliums, proteomics and *allium* s, metabolomics and alliums, metagenomics, and alliums. These articles were critically studied and used as references for the present review article. Our literature survey was not limited to specific time, area, institute, and authors, and thus, a comprehensive and unbiased review was ensured.

### Genomics

During the 19th century, the breeding approach, particularly in onions, was based only on mass selection. Further, the discovery of male sterility led to the development of F_1_ hybrids during the mid-20th century *(McCallum et al., 2008)*. Recently, hybrids, as well as open-pollinated varieties, have considerably increased onion productivity. However, overall, limited improvement is observed in *Allium* crops due to some inherent reasons, such as high heterozygosity, inbreeding depression, sexual sterility in garlic, clonal propagation, sensitivity toward photoperiod, and temperature. The whole-genome sequence assembly, which can be a crucial resource for functional genomics in *Allium* crops, is not yet available for any of the *Allium* members.

Recently, NGS platforms have been routinely used for the discovery of markers such as SSRs and SNPs. Baldwin et al. (2012a) developed advanced genomic SSR markers using NGS for the double dhaploid population; 921 SSRs were recognized from the genomic sequence of 6.6 Mbp. Whole-genome scanning methods have developed numerous SNPs in onion. Advanced genotyping technologies and doubled haploid in onion allowed frequent identification of SNPs *(Kim et al., 2014; Finkers et al., 2015; Shigyo, Fujito & Sato, 2019)*. The frequency of SNPs in onion is 1.7 SNPs/Kb, which is similar to that in other plant species. In massive onion germplasm, SNPs were validated, and the existing linkage map was mapped (Duangjit et al., 2013). A high-throughput genotyping approach, such as genotyping-by-sequencing (DArTseq), was utilized for the first time for diversity and structure analyses of a large garlic population with 417 accessions. These analyses have enabled in identifying unique and redundant accessions *(Egea et al., 2017)*. Jo et al. (2017) developed an onion genetic map by using the genotyping-by-sequencing approach. High-fidelity SNPs that satisfied the segregation ratio criteria were used for mapping the F_2_ onion population (NW-001 X NW-002). The map generated was 1383 cM in length,with a marker interval of 8.08 cM. Markers developed from the genic region have greater transferability than those from noncoding areas. Recently, the double digest restriction site-associated DNA sequencing (ddRAD-seq) approach was used for SNP discovery in inbred lines of Korean short-day onion. A total of 1904 SNPs were discovered and used in population structure analyses and genetic relationship studies *(Lee et al., 2018)*. Such SNPs or SSRs developed using NGS technologies can be helpful in developing precise linkage maps and managing genetic resources in onion as well as other related *Allium* species.

### Nuclear genome

Onion possesses a genome of 1C=16.75 pg, which is several times larger than the rice genome (1C=0.6 pg) (Bennet & Smith, 2002). Other *Allium* species also exhibit larger genome sizes ranging from 7.4 Gb in *Allium sibiricum* to 72.9 Gb in *A. validum (Shigyo, Khar & Abdelrahman, 2018)*. Among alliums, researchers have attempted sequencing the onion genome because of the economic importance of onions. Suzuki, Do & Mukai (2002) developed an onion BAC library consisting of 48,000 clones, which covered 0.39% of the genome. Kuhl et al. (2004) sequenced a normalized cDNA library yielding 18,388 sequences. The onion genome is low in GC content, and majority of the genome comprises repetitive sequences (75%–80%) *(Do, Suzuki & Mukai, 2004)*. Among plants, the gene density of onion is the lowest (1 gene/168 Kb), as confirmed by BAC-mediated and whole-genome shotgun sequencing projects. Pilot onion genome sequencing of two BACs revealed AT-rich (64.8%) sequences and long transposable elements, as evidenced in other larger plant genomes. Utilizing the methyl filtration technique potentially reduces unspecified and transposon sequences and produces approximately 10% higher gene encoding sequences (Jakše et al., 2008). The SEQUON project was recently initiated by Netherland to sequence the onion genome; under this project, four TrueSeq genomic DNA libraries were sequenced using IlluminaHiSeq 2500 *(Finkers et al., 2015)*. The resulted assembly was 10.8 Gb with 6.2 M contigs. Such a higher proportion of the genome coverage indicates the genome-wide distribution and sufficient divergence of ancient repeats, which can be assembled efficiently. In the present study, the authors propose that recently developed long-read sequencing platforms can further improve the assembly.

As mentioned earlier, majority of the *Allium* genome comprised repetitive sequences *(Do, Suzuki & Mukai, 2004)* that are composed of satellite DNA, tandem repeats, and transposable elements. A repeatome is the total quantity and types of repetitive DNA in the genome. The repeatome plays a crucial role in the evolution and organization of genomes (Maumus & Quesneville, 2014). Repeat DNA sequences in *Allium* crops were identified and mapped using cytogenetic techniques such as FISH (Kiseleva, Kirov & Khrustaleva, 2014; Mancia et al., 2015; Kirov et al., 2017). Hertweck (2013) performed comparative analyses of transposable elements from Asparagales and reported conservation as well as wide variation in the repeatomes of different taxa in Asparagales. Recently, repeatomes of few *Allium* species were studied using low-coverage genome sequencing by employing NGS and bioinformatic tools. More than 90% of the onion genome has been reported to be repetitive in nature *(Fu et al., 2019)*. Repeatomes of *A. cepa, A. sativum*, and *A. ursinum* were dissected using NGS technology,and the most prevalent repeat was Ty3/gypsy elements. Comparative analyses revealed that these repeats are significantly diverged in these species; hence, the authors could not detect common clusters during the analyses *(Peška et al., 2019*; https://www.ibp.cz/local/data/allium/). Studies on repeatome scan help in understanding speciation and karyotype evolution processes in *Allium* species. Further, knowledge of repeated DNA can be exploited for marker discovery and breeding in alliums.

Telomere sequences play a key role in the phylogenetic divergence of Asparagales. The last switch is responsible for the divergence of *Allium* from other genera. The omics approach was used to study telomeres in *Allium* species. *Fajkus et al.* (*2016*) used RNA-seq data and other techniques to characterize unusual telomeric sequences in *Allium* species. Recently, *Fajkus et al.* (*2019*) characterized the telomerase RNA of different species in Asparagales by using transcriptome and CRISPR/Cas-mediated genome editing (https://www.ibp.cz/ local/data/telomeraserna/). Interesting findings of these studies can facilitate the understanding of the functions of telomerase and telomere in the divergence of various taxa.

**Table 1:** List of mitochondrial and chloroplast genome sequence of *Allium* species.

Species	Genome	Platform	Length (bp)	GC %	References
*Allium cepa*	Mitochondria	NextSeq500	316,363	N.A.	*Kim et al. (2016)*
*Allium cepa*	Mitochondria	NextSeq 500	316,363 and 339,180	N.A.	Kim, Yang & Kim (2019)
*Allium cepa*	Mitochondria	GS FLX	316288	45.3	*Tsujimura et al. (2019)*
*Allium cepa*	chloroplast	454 FLX	153 538 and 153 355	36.8	*Von Kohn, Kiełkowska & Havey (2013)*
*Allium cepa*	Chloroplast	HiSeq2000	153,529, 153,440, and 153,568	N.A.	*Kim et al. (2015)*
*Allium* *victorialis*	Chloroplast	HiSeq2000	154,074	36.48	*Lee et al. (2017)*
*Allium prattii*	Chloroplast	Hiseq2000	154,482	37.02	*Jin et al. (2018)*
*Allium obliquum*	Chloroplast	HiSeq 1500	152,387	36.8	*Filyushin et al. (2018)*
*Allium sativum*	Chloroplast	HiSeq 1500	153,172	N.A.	*Filyushin et al. (2016)*
*Allium* *fistulosum*	Chloroplast	HiSeq 2500	153,164	36.8	Yusupov et al. (2019)
*Allium* *monanthum*	Chloroplast	Hiseq 2500	154,804	37	Xie et al. (2019a); Xie et al. (2019b)
*A. chrysanthum*	Chloroplast	Hiseq 2500	153,621	36.8	Xie et al. (2019a); Xie et al. (2019b)
*A. rude*	Chloroplast	Hiseq 2500	153,697	36.7	Xie et al. (2019a); Xie et al. (2019b)
*A. xichuanense*	Chloroplast	Hiseq 2500	153,673	36.7	Xie et al. (2019a); Xie et al. (2019b)
*A. chrysocephalum,*	Chloroplast	Hiseq 2500	153,710	36.8	Xie et al. (2019a); Xie et al. (2019b)
*A. maowenense,*	Chloroplast	Hiseq 2500	153,608	36.8	Xie et al. (2019a); Xie et al. (2019b)
*A. herderianum*	Chloroplast	Hiseq 2500	153,605	36.8	Xie et al. (2019a); Xie et al. (2019b)
*A*. *fistulosum*	Chloroplast	HiSeq 4000	153,162	36.8	*Huo et al. (2019)*
*A*. *tuberosum*	Chloroplast	HiSeq 4000	154,056	36.9	*Huo et al. (2019)*
*A*. *sativum*	Chloroplast	HiSeq 4000	153,189	36.7	*Huo et al. (2019)*
*A*. *cepa*	Chloroplast	HiSeq 4000	153,586	36.8	*Huo et al. (2019)*
*Allium* *ovalifolium*	Chloroplast	HiSeq 4000	153,635	37	*Sun et al. (2019)*

### Mitochondrial genome

Researchers have recently completed the sequencing of the mitochondrial genome of onion varieties (Table 1); the data obtained can help in understanding the male sterility mechanism in onion and its further use in breeding programs. The mitochondrial genome of onion (316 kb) containing the CMS-S male-sterile cytoplasm was sequenced using the Illumina NextSeq500 platform. They also identified *cox1* as part of chimeric *orf725*, which is one of the candidate genes for CMS *(Kim et al., 2016)*. The mitochondrial genome of the CMS-S-type onion variety “Momiji-3” was characterized which have a multi-chromosomal structure resulting from recombination events. Examination of transcript data revealed RNA editing at 635 positions, and a candidate gene for CMS in “Momiji-3” was also found to be *orf725 (Tsujimura et al., 2019)*. Similarly, Kim, Yang & Kim (2019) compared the mitochondrial genome of two recently diverged cytoplasms—male-fertile and male-sterile CMS-T-like cytoplasms and obtained almost identical sequences. They also identified a chimeric gene, *orf725*, as a candidate gene for CMS. The mitochondrial genome of more *Allium* species with different cytoplasms should be sequenced for an improved understanding of the mitochondrial genome.

### Chloroplast genome

The chloroplast genome is known for its conserved nature, maternal inheritance, and collinear gene order. Therefore, the chloroplast genome is widely used for phylogeny and diversity analyses in plants. The chloroplast genome of several *Allium* species have been characterized using NGS platforms *(Filyushin et al., 2018; Xie et al., 2019a; Yusupov et al., 2019)* and used for marker discovery *(Lee et al., 2017; Kim, Park & Yang, 2015)*, phylogenetic analyses *(Xie et al., 2019b; Huo et al., 2019)*, and the study of male sterility (Von Kohn, Kiełkowska & Havey, 2013; Kim, Park & Yang, 2015; Table 1). The complete chloroplast genome is 152,387–154,482 bp in length,with a GC content of 36%–37% *(Filyushin et al., 2016; Lee et al., 2017; Xie et al., 2019a; Xie et al., 2019b; Yusupov et al., 2019)*. Minor variations in the length of the chloroplast genome, among the *Allium* species, might be due to the occurrence of indels. The number of genes in this genome ranges from 114 to 141. Repeat analyses revealed that all chloroplast genomes contain similar repeat sequences *(Huo et al., 2019)*. Information generated from chloroplast genome sequencing could be harnessed for studying and improving *Allium* crops.

### Transcriptomics

**Figure 2:**
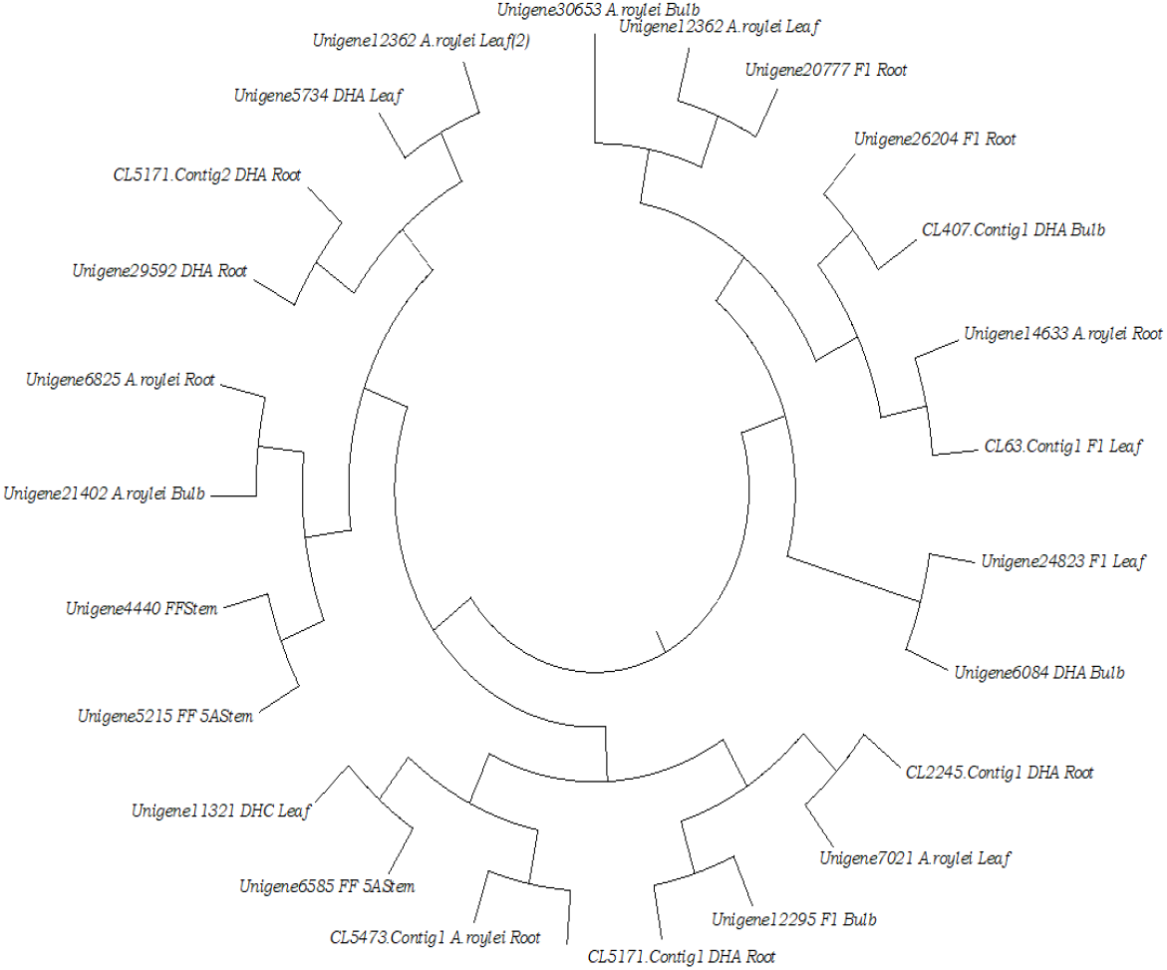
Inferred Ancestral Sequences of WRKY4 like sequences from AlliumTDB.

Transcriptomes are being routinely studied using the RNA-seq approach because of the affordability of sequencing and availability of analyses pipelines for extracting meaningful and useful information. Transcriptomes facilitate transcript profiling of a tissue at a specific stage or time and enable validation and annotation of these putative differentially expressed transcripts. Sun et al. (2013) and Baldwin et al. (2012b) performed transcriptome characterization in garlic and doubled haploid onion respectively for the first time. Thereafter, several published articles have reported the use of RNA-seq for studying organ development, male sterility, marker discovery, abiotic stress response, and flavonoid synthesis in various *Allium* species (Table 2). Kim et al. (2015) attempted structural annotation of onion RNA-seq by using an integrated structural gene annotation pipeline. Similarly, Sohn et al. (2016) developed a draft reference transcript for onion by using long-read sequencing technology, suggesting that further progress in sequencing chemistry will help in whole-genome sequencing in alliums*.* Kazusa DNA Research Institute and Yamaguchi University, Japan, have developed a transcriptome database for *Allium* species—“AlliumTDB” (http://alliumtdb.kazusa.or.jp/). This database harbors transcriptome data of 12 different libraries of root, stem, bulb, and leaves of different species and their doubled haploids. It is a vital resource of genetic information in *Allium* crops. The desired information can be retrieved from the “KEYWORD” page (http://alliumtdb.kazusa.or.jp/keyword.html) by searching the keywords as genes, TFs etc. This key word search results into various available information on the searched terms from many databases like TAIR, NCBI NR, and RAP-DB. To discriminate sequences of transcription factors in AlliumTDB, we searched for the important transcription factors like WRKY (645), AP2/ERF (863), NAC (735), Myb (1970), DOF (293), HSF (256), ARF2 (123), ARF1 (95), LFY (6), C2H2-type zinc finger (1193), Basic-leucine zipper (759), GATA type Zinc finger (405) etc. The results have been summarized in to File S1 having the set name of unigene (DB), sequence name of unigene (AC), % identity (Iden) and unigene length (Annotation) etc. The sequences of WRKY4 TFs were filtered and aligned on Mega6 Software (Tamura et al., 2013) using Maximum Likelihood method (Nei & Kumar, 2000) under the JTT matrix-based model (Jones, Taylor & Thornton, 1992). The sequence diversity of all the sequences from *A. cepa* transcripts is presented in Fig. 2.

**Table 2:** List of the RNA seq analyses performed in *Allium* species.

**Species**	**Platform**	**Tissue**	**Purpose/observation**	**Reference**
*Allium sativum*	Illumina HiSeq 2000	Dormant and sprouting bud	Discovery of genes in sulfur assimilation	*Sun et al. (2012)*
*Allium sativum*	Illumina HiSeq 2500	Whole plant of 10 and 45 days old	Developed SSR marker in garlic and studied their transferability to other *Allium* species	*Liu et al. (2015)*
*Allium sativum*	MiSeq v3 platform	Leaves, basal plate, roots inflorescence, flowers, cloves	Organ specific transcriptome for identification key gene and mechanism of organ development	*Kamenetsky et al. (2015)*
*Allium sativum*	Roche 454-FLX	Leaves, pseudostems, and roots	SNPs and indels discovery	*Havey & Ahn (2016)*
*Allium sativum*	Illumina HiSeq 2000	Flower buds of male sterile and fertile lines	Energy deficiency of tapetum cells might be reason of male sterility in garlic	*Shemesh-Mayer et al. (2015)*
*Allium sativum*	Illumina HiSeq™ 2000	Shoot apex	Genes differentially expressed in shoot apex were identified	*Sun et al. (2013)*
*Allium cepa*	Illumina HiSeq 2500	Leaf	Discovery of NAC transcription factor in onion	*Zheng et al. (2016)*
*Allium cepa*	Illumina HiSeq 2000	Outer, intermediate and inner scale	Programmed cell death in onion skin formation	*Galsurker et al. (2017)*
*Allium cepa*	Illumina HiSeq 2000	Bulb	ISGAP was used for higher accuracy of transcript annotation	*Kim et al. (2015)*
*Allium* *cepa*	Roche 454 FLX platform	Bulbs, tissue from leaves, unopened umbels, bulbs, and roots	SNP development based on saturated map	*Duangjit et al. (2013)*
*Allium cepa*	Illumina HiSeq 2000	Leaf	Differentially expressed genes were identified at freezing temperature and development of SSR and SNPs markers	*Han et al. (2016)*
*Allium cepa*	HisSeq™ 2500	Bulb	Dissected sucrose metabolism during bulb formation	*Zhang et al. (2016)*
*Allium cepa*	Illumina HiSeq™ 2000	Anthers at the tetrad stage	Identification of differentially expressed gene in anthers of sterile and maintainer line	*Yuan et al. (2018)*
*Allium cepa*	Illumina HiSeq 2000	Bulb	The *AcPMS* 1 involved in DNA mismatch Repair, is the best candidate for fertility restoration	*Kim et al. (2015)*
*Allium cepa*	454™ GS-FLX	Leaves and shoot	Developed toolkit for bulk PCR marker designing from transcriptome data	*Baldwin et al. (2012b)*
*Allium cepa*	Illumina HiSeq 2000	Scales	Studied differential response of outer and inner scales to the heat treatment	Galsurker et al. (2018)
*Allium cepa*	Illumina HiSeq 4000	Bulb	Variation in bulb colour	*Zhang et al. (2018)*
*Allium cepa* and interspecific hybrids	Illumina HiSeq2000	Leaves	Develoment of SNP markers from interspecific hybrids for introgression breeding	Scholten et al. (2016)
*Allium cepa*	PacBio_RSII platform, P4-C2 chemistry	Flowers, leaves, bulbs and roots	Long read sequencing was used to construct a draft reference transcripts	*Sohn et al. (2016)*
*Allium porrum*	Illumina HiSeq 2500	Leaves	Identification of *CONSTANS*-like genes which play role in flowering	Liu et al. (2018)
*Allium porrum*	Illumina MiSeq	Leaves and cloves	Elucidation of organosulfur metabolic pathway	*Mehra et al. (2019)*
*Allium fistulosum*	Illumina HiSeq 2500	Bulb	Study of saponin biosynthetic pathway and their possible role in *fusarium* resistance	*Abdelrahman et al. (2017)*
*Allium fistulosum*	Illumina HiSeq 2000	Leaves, false stem, basal plate and root	Gene for sulfur and selenium metabolism were identified, SSR marker were developed	Sun et al. (2016)
*Allium fistulosum*	GS-FLX and HiSeq 2000	2-week-old seedlings, leaf, roots, basal meristem, immature flower bract, mature bract, opened flowers, immature fruits and sliced pseudostem	EST markers were developed for mapping and these marker will enable comparison with bulb onion	*Tsukazaki et al. (2015)*
*Allium fistulosum*	Illumina HiSeq 2000	Leaves	Identified four genes for wax content and developed 1,558 SSR markers	*Liu et al. (2014)*
*Allium fistulosum*	Illumina HiSeq 2000	Inflorescences	Differential gene expression in CMS and maintainer line	*Liu et al. (2016)*
*A. fistulosum,* shallots and 8 monosomic addition lines	HiSeq 2500	Bulbs	Found hot spot for flavonoid synthesis on chromosome 5A	*Abdelrahman et al. (2019)*
*Allium tuberosum* Rottler ex Spr	Illumina HiSeq 2000	Leaves, shoots and roots	Performed gene annotation and SSR identification in Chinese chive	*Zhou et al. (2015)*
*A. sativum, A. porrum, A. tuberosum, A.* *Fistulosum, A. ascalonicum, A. cepa, A. cepa var. agrogarum, A. chinense, A.* *macrostemon*	Illumina HiSeq 2500	Leaf	PCD might involve in development of fistular leaves	*Zhu et al. (2017)*
*A. ursinum, A. cepa, A. angulosum, A. cernuum, A. ericetorum, A.* *fistulosum, A. nutans, A. sativum, A. scorodoprasum, A. senescens* and *A. vineale*	Illumina HiSeq 2000	Leaves and roots	Reported that the unusual telomeric sequence present in *Allium* species, demonstrated the synthesis of telomere by telomerase	Fajkus et al. (2016)
*A. nutans, A. cepa, A. ursinum, A. angulosum, A.* *ericetorum, A. fistulosum, Tulbaghia violacea, Scilla peruviana,* and *Cestrum elegans*	Illumina NextSeq500	Leave sand root	Studied telomeres in order Asparagales	Fajkus et al. (2019)

### Marker discovery

The limitation of DNA markers is one of the hurdles in the breeding of*Allium* crops and their improvement *(Chinnappareddy et al., 2013*; Khosa et al., 2016a; Khosa et al., 2016b). The RNA-seq approach has been widely used in the discovery of microsatellites SSRs and SNPs in several species. In alliums, few attempts have been made to develop markers from transcriptome data and linkage maps. Baldwin et al. (2012a) and Baldwin et al. (2012b) performed RNA-seq of the doubled haploid bulb onion “CUDH2150” and the genetically distant “Nasik Red” by using 454™ sequencings, and the mapping of reads revealed 16836 indels and SNPs. These markers have been further used for developing a linkage map of more than 800 cM covering all marker linkage groups. Transcriptomes of two inbred lines of onion were sequenced on the Roche-454 platform, and 3364 SNPs were identified on 1716 cDNA contigs. These SNPs were further used in the genetic mapping of different mapping populations *(Duangjit et al., 2013)*. RNA-seq was used for identifying SNPs from male-fertile and male-sterile onion genotypes. From 141 contigs, 430 homozygous SNPs were identified and further used for identifying candidate genes for male sterility *(Kim et al., 2015)*. Similarly, Shigyo, Fujito & Sato (2019) highlighted the use of RNA-seq-derived SNP markers in the development of high-density maps. A garlic transcriptome was sequenced using the Illumina platform that yielded 135360 unigenes. The EST data were used for developing 1506 SSR markers *(Liu et al., 2015)*.

Similarly, microsatellite, SNP, and indel markers were discovered from the transcriptome data of garlic *(Havey & Ahn, 2016; Chand et al., 2015*). *Tsukazaki et al. (2015)* used the transcriptome shotgun assembly and Illumina HiSeq 2000 for RNA-seq analyses in *A. fistulosum*. Analyses of 54904 unigenes led to the discovery of 2396 SSRs, 9002 SNPs, and 4335 indels. These markers were used in the genetic mapping of *A. fistulosum* in comparison with bulb onion due to the availability of common markers. Similarly, EST-SSR markers were developed for Welsh onion (Yang et al., 2015) and Chinese chive *(Zhou et al., 2015)* from their respective transcriptome data.

### Male sterility

Cytoplasmic male sterility is one of the most crucial traits of *Allium* crops and is routinely exploited for hybridization. However, the mechanism of male sterility is not well characterized at the molecular level in alliums. In onion, several molecular markers have been developed for selecting CMS genotypes. Kim et al. (2015) used BSA and RNA-seq analyses to identify candidate genes for restoring fertility. Several SNPs that differentiate male-sterile and male-fertile bulks were identified, along with 14 contigs that showed their perfect association withthe *Ms* locus. Among these contigs, *AcPMS1* involved in the DNA repair mechanism is the best candidate gene and the most reliable marker for selecting the restorer line. Further, the transcriptome of the tetrad stage anther from male-sterile and maintainer lines was analyzed. Five genes that are associated with male sterility in onion were identified, of which two were cytoplasmic (*atp9* and *cox1*) and three were coded by the nucleus (*SERK1*, *AG,* and *AMS*) *(Yuan et al., 2018)*. Differential gene expression in male-sterile and maintainer lines of Welsh onion was investigated using the transcriptome approach, and more than 4000 unigenes were found to be differentially expressed, including genes known for their role in male sterility, such as *F-type ATPase , NADH dehydrogenase , and cytochrome c oxidase (Liu et al., 2016)*. These studies have revealed that CMS in alliums is governed by both mitochondrial as well as nuclear genes and the interaction between them. The male-sterile garlic genotype showed tapetal hypertrophy, which is a cause of sterility. Integrated transcriptome and proteome analyses revealed several differentially expressed genes and proteins in male-sterile and fertile genotypes. These candidate genes (*AP3*, *ms2*, *mmd1, gpat2*, *nad7*, *ccmC*, *cox2, 18S rRNA, flavanol synthase ,* and *sod*) were involved in male sterility in garlic. Physiological and molecular analyses indicated that respiratory restriction or non-regulated programed cell death (PCD) might result in energy deficiency, and in turn, sterile pollens *(Shemesh-Mayer et al., 2015)*.

### Abiotic stress

Plants are consistently subjected to various types of environmental stress because of their sessile nature and due to weather uncertainty in the field. This stress is responsible for significant yield reduction and economic loss of up to 50% *(Atkinson & Urwin, 2012)*. Because climate change affects crops, we need to develop stress-tolerant and climate-smart varieties of crops. Roots of *Allium* crops are shallow, with a maximum root depth of 0.18 meter, and thus, a slight deepening of the water level renders onion prone to drought stress *(Drinkwater & Janes, 1955)*. Flooding, waterlogging, and salinity severely affect the yield of *Allium* crops *(Yiu et al., 2009; Sta-Baba et al., 2010)*.

The abiotic stress response in *Allium* crops has not been studied in detail; RNA-seq has enormous potential in elucidating these stress response mechanisms. The shallot is well adapted to abiotic stress compared with onion *(Currah, 2002)*. Differential transcriptomic and metabolomics analyses were performed in the doubled haploids of onion and shallots. Genes involved in amino acid, osmoprotectant, and flavonoid biosynthesis were significantly upregulated in the doubled haploids of shallot *(Abdelrahman et al., 2015)*. These genes might play a role in improved adaptability of shallot to abiotic stress, and thus, they may be used as candidate genes in onion breeding for stress tolerance. The response of cold-tolerant and -susceptible onion genotypes was studied using a transcriptomic approach at cold and freezing temperatures. A total of 491 genes were differentially expressed at freezing temperatures among cold-tolerant and susceptible onions *(Han et al., 2016)*. These genes need to be explored further for better adaptation of onion crops growing at high altitudes to low temperature. Dufoo-Hurtado et al. (2013) reported the upregulation of phenolic metabolism-related genes in garlic after low-temperature conditioning of “seed” cloves. NAC (NAM, ATAF, and CUC) transcription factors play an essential role in plant stress response as well as development. Zheng et al. (2016) identified 39 NAC genes from onion leaves by using RNA-seq and classified them into five groups of NAC transcription factors.

### Biotic stress

Majority of alliums constitute economically important cash crops, and significant losses occur due to diseases and pests in these crops. Agrochemicals applied to manage biotic stress lead to an increase in the cost of production, ultimately reducing the income of farmers. There are minimal resistance genes available in *Allium* crops. RNA-seq can prove to be an excellent tool for discovering novel genes for pest and disease resistance in crops such as *Allium*, where the draft genome sequence is not available. Till date, no single report has published on transcriptome analyses in alliums concerning biotic stress responses. In onions, limited resistance sources are available, and therefore, related or wild species need to be explored for disease resistance . Scholten et al. (2016) performed transcriptome analyses of interspecific hybrids of *A. roylei* and *A. fistulosum* and onion varieties. SNP markers developed from these data were used for linkage and QTL mapping. QTL for resistance to *Botrytis squamosa* was identified on the 6th chromosome of *A. roylei*. Steroidal saponins are involved in plant defense against different biotic stress. Abdelrahman et al. (2017) isolated the saponin compound called alliospiroside A from the monosomic addition lines of *A. fistulosum* and reported its antagonistic activity against *Fusarium*. RNA-seq analyses of these monosomic lines and normal lines led to the identification of 50 unigenes for saponin biosynthesis and found the upregulation of these genes in the monosomic addition line of *A. fistulosum*. Cuticlar and epicuticular wax depositions on onion leaves are known to involve in biotic stress tolerance, especially against onion thrips *(Damon, Groves & Havey, 2014; Silva et al., 2015)*. RNA-seq analyses of a waxy and non-waxy mutant of Welsh onion revealed differential expression of 798 genes. Four of these genes were validated using qPCR, and COG annotation revealed that they were involved in lipid biosynthesis and defense response in plants *(Liu et al., 2014)*. Such studies might help in understanding wax deposition in other *Allium* species and thus in developing pest- and disease-resistant varieties. Another report on garlic reported the identification of the presence of four known garlic allexiviruses; A, C, E, and X from transcriptome data *(Kamenetsky et al., 2015)*. Thus, transcriptome analysis is a highly sensitive method for identifying known and unknown viruses infecting *Allium* crops. It can be used for studying plant–virus interaction in a tissue-specific manner. Mohapatra & Nanda (2018) identified chitinase genes from transcriptome data by using an *in silico* approach. Similarly, *RNA-seq* data deposited in databases could be mined for the discovery of genes involved in plant defense response.

### Organ development

Organ development in plants is a highly organized and precisely regulated process in response to indigenous signals as well as environmental cues. Flowering and bulb development is a critical process in *Allium* crops. Gene regulation plays a vital role in a smooth transition of growth phases in the life cycle of the plant; however, little information is available about gene interaction and regulation. Expression analyses using transcriptome has enabled extraction of this information for a large number of genes at the global level in less time and at reduced costs. Therefore, in non-model crops, such as alliums, NGS-based expression analyses helped in understanding the mechanism of plant development.

Transcriptome analyses of dormant and sprouting garlic shoot apices were performed to elucidate differential gene expression and reveal the role of genes in dormancy as well as sprouting in garlic. More than 20000 unigenes were found to be upregulated in the sprouting shoot apex. Several candidate genes (*ENHYDROUS, DAG1, DAM, DTH8*, etc.) involved in dormancy and sprouting of the shoot apex were identified (Sun et al., 2013). Kamenetsky et al. (2015) developed an organ-specific transcriptome catalog for fertile garlic*.* Different organs exhibited a variation in gene expression profiles, and the highest number of organ-specific reads were reported from the flower. Genes in organosulfur metabolism exhibited an organ-specific profile, whereas several genes involved in flowering and bulb formation were also expressed in leaves, indicating the role of signal transduction. Flowering is a major challenge in garlic breeding, and thus, such studies in fertile garlic will open avenues for understanding the genetic mechanism underlying the reproductive process in garlic as well as other alliums. The *CONSTANS* and *CONSTANS -like* (*COL*) genes are known for their prime role in flowering. Transcriptome analyses of leek led to the identification of 17 putative *ApCOL* genes, some of which exhibited high similarity with *COL* genes from other species involved in the modulation of flowering and heading date *(Liu et al., 2018)*. Different leaf morphologies are observed in alliums, such as flat, solid, and fistular. Cellular studies have revealed that fistular leaves arise from a solid precursor by PCD *(Ni et al., 2015)*. A molecular evidence for this finding was provided using transcriptome analyses of nine important species of *Allium* with different leaf morphologies *(Zhu et al., 2017)*. Phylogenetic analyses of transcriptome revealed that genes associated with PCD presented rapid diversification in fistular leaf species or showed conserved nature in solid leaf species in evolutionary history. Several potential genes involved in plant PCD were subjected to positive selection or evolved in species having fistula-type leaves. This varying selection pattern of PCD-related genes might play an essential role in the development of cavities in fistular leaves. *FLOWERING LOCUS T* (*FT*) is the major component of the floral signal molecule as well as other plant development processes. Transcriptome analyses of the doubled haploid onion led to the discovery of six *FT*-like genes that play a role in flowering and bulb formation. *AcFT2* promotes flowering, whereas *AcFT1* favors bulb formation, and *AcFT4* is antagonistic to *AcFT1 (Lee et al., 2013)*. These functions of *AcFT* genes were further confirmed through transgenic studies in *Arabidopsis*. Thus, *FT*-like gene regulation should be studied in non-flowering and non-bulb-forming alliums and their wild relatives for a further in-depth understanding of their role in alliums. Khosa et al. (2016a) and Khosa et al. (2016b) performed RNA-seq analyses for the doubled haploid bulb onion “CUDH2107” from leaves, various stages of flowering, and roots. They identified several genes involved in flowering and male fertility in onion, which are the orthologs of rice genes. Sucrose metabolism during onion bulb development is a crucial phenomenon *(Mallor et al., 2011)*. Zhang et al. (2018) identified key genes expressed during sucrose metabolism through transcriptome analysis. They performed RNA-seq analyses in three bulb stages (15, 30, and 40 days after bulb swelling), and KEGG analyses revealed that “sucrose and starch metabolism”-related genes were dominant. Expression of the sucrose transporter was the highest during early stages. Sucrose synthase and invertase seem to be involved in sucrose breakdown. Fructose and glucose contents gradually increased from early to later stages, whereas sucrose content decreased. Sucrose metabolism and bulb growth suggested that 30–40 DAS is a period of rapid expansion of bulb (Zhang et al., 2018). . Desiccation and senescence processes lead to the formation of the onion bulb skin. The bulb skin plays a crucial role in the storage and shelf life of the onion bulb and influence its consumer acceptance *(Chope et al., 2012)*. Scanning electron microscopy revealed that the desiccation of the outer scale occurs from inside out, and DNA degradation indicated the critical role of PCD in skin formation. Gene ontology enrichment of transcripts from outer, intermediate, and inner scales revealed an increase in processes related to defense response, PCD processes, carbohydrate metabolism, and flavonoid biosynthesis in the outer scale, whereas the inner scale exhibited increased metabolism and developmental growth processes *(Galsurker et al., 2017)*. These results indicated that the metabolism for bulb skin formation occurs only in the outer scales of onion.

### Flavonoids and bulb color

Bulb color is one of the important quality traits in onion. Various bulb colors exist, such as white, yellow, red, pink, chartreuse, and gold. Variation in this trait is governed by mutations in structural and regulatory genes of the flavonoid biosynthesis pathway (Khandagale & Gawande, 2019). Baek, Kim & Kim (2017) performed RNA-seq and bulk segregant analyses of yellow and F_2_ white plants to develop a marker linked to the C locus that determines the white bulb color in onion. Ninety-seven genes, including previously identified genes of the flavonoid pathway, exhibited more than five-fold expression in the yellow bulk.

Similarly, regulatory genes for the MBW complex were upregulatedin the yellow bulk. SNPs in the gene coding for glutathione S-transferase revealed linkage with the C locus. Transcriptome analyses of white and dark red onions led to the identification of 16 DEGs that play a critical role in the flavonoid biosynthesis pathway. Flavonoid 3′, 5′-hydroxylase (*F3*′5′*H*) and dihydroflavonol 4-reductase (DFR) genes play crucial roles in the biosynthesis of dark red bulbs. Further, the ratio *F3*′5′*H*: *F3*′*H* plays an essential role in the diversity of bulb color *(Zhang et al., 2018)*.

### MicroRNAs

MicroRNAs (miRNAs) are a group of small, endogenous noncoding RNAs (ncRNAs) comprising approximately 18–25 nucleotides that regulate gene expression in animals, plants, and protozoans. miRNAs control approximately 60% protein-coding gene activities and regulate numerous cellular processes *(Bartel, 2009)*. miRNAs regulate gene expression through translational repression or target mRNA degradation *(Chekulaeva & Filipowicz, 2009)*. Among *Allium* crops, two reports have reported discovery of miRNA in garlic and onion by using the *in silico* approach (Panda et al., 2014; Kohnehrouz, Bastami & Nayeri, 2018). *Fusarium oxysporum f. sp. cepae* (FOC) is the most devastating pathogen that infects roots and basal plates of garlic. (Chand et al., 2017) performed small RNA sequencing of a FOC-resistant line after infection and discovered 45 miRNAs responsive to FOC. Overexpression of *miR164a*, *miR168a*, and *miR393* resulted in decreased fungal growth as well as upregulation of defense genes. Similarly, in an earlier study, Chand, Nanda & Joshi (2016) reported that miR394 functions as a negative modulator of FOC resistance in garlic. miRNA thus plays a huge role in the regulation of genes in *Allium* crops in response to stress as well as development, and thus, for its application in crop improvement, more miRNAs and their targets need to be discovered. Recently, we performed genome-wide identification of miRNA in *Thrips tabaci*, along with their expression profiling and target prediction *(Balan et al., 2018)*. These miRNAs and their targets can be used for the management of onion thrips by using RNA interference technology.

### Proteomics

Proteomics is the high-throughput study of proteins expressed in specific tissues in an individual at a specific time or developmental stage. It plays a key role in interactive or meta-omics analyses, as it complements gene expression and metabolomic studies and hence is more helpful in crops, such as alliums*,* where limited genomic information is available (Table 3). Proteome alteration in sprouts after low-temperature conditioning of garlic cloves was studied *(Dufoo-Hurtado et al., 2015)*. Differentially synthesized proteins were analyzed using 2-DE and LC-ESI-MS/MS and were found to be involved in various biological processes. This study complemented other studies reporting changes in physiology, biochemistry, and molecular biology of cloves after low-temperature treatment *(Dufoo-Hurtado et al., 2013; Guevara-Figueroa et al., 2015)*. Proteomic changes during freeze–thaw injury and recovery in onion scales were studied. Majority of injury-related proteins (IRPs) were found to be antioxidants, stress proteins, molecular chaperones, and proteins of energy metabolism; these IRPs were induced as a first response to mitigate injury. Recovery-related proteins involved in injury repair facilitates cellular homeostasis, cell wall remodeling, reactive oxygen species scavenging, defense against possible post-thaw infection, and regulating the energy budget to sustain these processes *(Chen et al., 2013)*. Plant’s ability to recover from freeze–thaw injury is a critical factor contributing to tolerance to this stress. Similarly, proteome level changes in response to toxic metals, such as copper and sodium selenite, were also reported in onion (*Qui et al., 2015; Karasinski et al., 2017)*. 2-DE and MS approaches identified 47 differential abundant protein spots between lower and upper epidermis of scales in red and yellow onions; among these, 31 were reported to be unique proteins. These differential proteins were involved in flavonoid synthesis, response to stress, and cell division *(Wu et al., 2016)*. While studying gametogenesis and sterility in garlic, significant differences were observed in the 2-DE profile between sterile and fertile genotypes. A variation was observed in protein maps of different stages of microsporogenesis *(Shemesh-Mayer et al., 2013)*. These differential proteins need to be investigated using MS to elucidate their identity and potential role in fertility.

**Table 3:** List of the proteomics analyses performed in *Allium* species.

**Species**	**Organ/tissue**	**Platform**	**Trait/aim**	**Reference**
*Allium sativum* L	Garlic sprouts	2-DE, LC-ESI-MS/MS	Low temperature conditioning alters the proteome	*Dufoo-Hurtado et al. (2015)*
*Allium cepa* var. agrogarum	Roots	2-DE, AutoFlex TOF/TOF II	Proteomic response of roots to Cu stress	Qin et al. (2016)
*Allium sativum* L	Bulbs	2-DE, XCT mass spectrometer	Characterizatio of Copper–Zinc Superoxide dismutase	*Sfaxi et al. (2012)*
*Allium cepa L*	Scale epidermis	2-DE, MALDI-TOF/TOF	Studied differential protein abundance in upper and lower epidermis	*Wu et al. (2016)*
*Allium cepa L*	Scales	2-DE, 4700 MALDITOF/TOF	Studied the proteomic response during and after recovery of freeze-thaw injury	*Chen et al. (2013)*
*Allium sativum L*	Anthers	2-DE	Study revealed proteomic differences between fertile and sterile genotypes, developmental stages	*Shemesh-Mayer et al. (2013)*
*Allium cepa L*	Roots	nanoLC-ESI-QOrbitrap-MS	Studied the effect of sodium selenite at proteome level	*Karasinski et al. (2017)*

### Metabolomics

Plants produce large numbers of metabolites of diversified structures and physical properties in varying abundance. These metabolites vary with the genotype, plant development, environmental conditions, storage, and processing. Metabolite profiling has now been implemented on a broad range of plant species, including but not limited to, tomato, potato, rice, wheat, strawberry, *Medicago*, *Arabidopsis* and *Allium* species**.** Approximately, 13000 onion accessions are held in gene banks worldwide (Böttcher et al., 2017), but only a small portion of this diverse *Allium* resource is exploited due to the paucity of information (Soininen et al., 2014a; Soininen et al., 2014b; Soininen et al., 2012)**.** *Allium* crops are extensively consumed for their broad array of health-promoting effects, which are assigned to the presence of different profiles of organosulfur compounds (OSCs), such asalk(en)yl cysteine sulfoxides, S-allyl cysteine, thiosulfinates (mainly allicin), diallyl sulfides, vinyldithiins, and (E)- and (Z)-ajoene (Block et al., 2010; Ramirez et al., 2017). Metabolites present in *Allium* species may represent their adaptation to different environmental cues during the domestication process. They also govern important characters in alliums, such as color, pungency, taste, stress tolerance, and medicinal properties. A wide range of modern techniques, such as Liquid Chromatography-Mass Spectrometry (LC–MS), Gas Chromatography–MS (GC-MS), Nuclear Magnetic Resonance (NMR), LC-Fourier Transform Ion Cyclotron Resonance (FTICR)-MS, LC-tandem quadrupole (QqQ)-MS, LC-Quadrupole Time-of-Flight (QTOF)-MS/MS, and Ultrahigh Performance Liquid Chromatography-coupled Electrospray Ionization Quadrupole Time-of-Flight Mass Spectrometry (UHPLC/ESI-QTOFMS), have been used to explore, determine, and characterize the metabolic profile of *Allium* species (Soininen et al., 2014a; Soininen et al., 2014b; Soininen et al., 2012; Nakabayashi et al., 2016; Böttcher et al., 2017; Molina-Calle et al., 2017; Hrbek et al., 2018; Table 4).

**Table 4:** List of the metabolomic analyses performed in *Allium* species.

**Species**	**Organ/tissue**	**Platform**	**Trait/aim**	**Reference**
*Allium sativum*	Clove	LC-MS/MS	Low temperature conditioning led to high phenolic and anthocyanin content	*Dufoo-Hurtado et al. (2013)*
*Allium cepa L.*	Bulb	UHPLC/ESI-QTOFMS	Variation in metabolite profiles of bulbs due to genetic and environmental factors	*Böttcher et al. (2018)*
Finnish onions,, German long shallot, French leek and Chinese garlic	Bulb	NMR and HPLCMS	Quantification of metabolite in *Allium* species	Soininen et al. (2014a)Soininen et al. (2014b)
*Allium sativum, Allium cepa L.*	Dried bulbs	GC/MS, UPLC/MS	Effect of drying methods on chemical composition was investigated	*Farag et al. (2017)*
*Allium sativum* L.	Cloves	DART-HR-OrbitrapMS, HPLC-ESI-HR-TOFMS	Authenticity of garlic from different geographical locations was assessed	Hrbek et al. (2018)
*Allium sativum* L		GC/MS, HPLC-MS/MS	Developed rapid, simple and efficient method for sensory evaluation of garlic	*Liu et al. (2019)*
*Allium cepa* L.	Fresh and stored bulbs	NMR	Highlighted the use of Metabolomics for food authentication	*Saviano et al. (2019)*
*Allium cepa* L.				*Farag et al. (2019)*
*Allium cepa*	Bulb	LC/ESI-QTOFMS	Metabolic profiling of onion cultivars	*Böttcher et al. (2017)*
35 *Allium* species	Bulb and leaves	HPLC-ESI-HRMS and NMR	Identification and quantification of metabolite with *α*-glucosidase inhibitory activity	Schmidt, Nyberg & Staerk (2014)
*Allium sativum*	Cloves	LC–QTOF MS/MS	Analysed the differential composition of fresh and black garlic	*Molina-Calle et al. (2017)*
*Allium cepa, Allium sativum*, *Allium fistulosum*	Bulbs	LC-FTICR-MS	Performed chemical assignment of structural isomers of S-containing metabolites	*Nakabayashi et al. (2016)*
*Allium sativum*	Bulbs	HPLC and LC-FTICR-MS	Sulphur containing metabolites were analysed	*Yoshimoto et al. (2015)*
*Allium cepa*	Bulbs	^1^H NMR and LC–MS/MS	Bioactive compounds were identified	*Soininen et al. (2012)*
*Allium porrum* L	bulbs	^1^H NMR and HPLC–MS	Demonstrated metabolome profile of different varieties	Soininen et al. (2014a)Soininen et al. (2014b)
*Allium roylei*	Leaves, bulbs, and roots-basal stems	HPLC	Bioactive compounds from different organs were analysed	*Abdelrahman et al. (2014)*
*A. fistulosum,* shallots and 8 monosomic addition lines	Bulbs	LC-QqQ-MS	Metabolites in flavonoid pathway were studied	*Abdelrahman et al. (2019)*

Among alliums, metabolic profiling is so far performed in onion bulbs, considering cultivars and cultivation year *(Böttcher et al., 2018)*; change in organosulfur metabolites after heat treatment in onion (Kim et al., 2016); differentiation profiling of black and fresh garlics *(Molina-Calle et al., 2017)*; low-temperature conditioning of “seed” cloves *(Dufoo-Hurtado et al., 2013)*; volatile profiling of onion and garlic after subjecting to different drying methods *(Farag et al., 2017)*; metabolic profiling of leaves, bulbs, roots, and basal stems in *A. roylei* and *A. porrum* L. *(Abdelrahman et al., 2014; Soininen et al., 2014a; Soininen et al., 2012*); authentication of the geographic origin of garlic *(Hrbek et al., 2018)*; bulb color and flavonoids *(Zhang et al., 2018; Abdelrahman et al., 2019)*; and metabolic profiling of fresh and stored onion bulbs *(Saviano et al., 2019)*. Metabolomics, along with the artificial neural network, was used for sensory evaluation of garlic and garlic products by using 89 quality indicators *(Liu et al., 2019)*. Farag et al. (2019) studied the effect of the pickling process on onion metabolome and reported a decrease in secondary metabolites; thus, pickling using brine negatively affects the health benefits of onion. Functions and application of some metabolites are presented in Table S2. Onions produce discrete metabolites, including fructo-oligosaccharides (Galdón et al., 2009), OSCs (Block et al., 2010), and flavonoids *(Olsson, Gustavsson & Vagen, 2010)*. In plants, flavonoids are present as flavonoid aglycones, flavonoid O-glycosides, flavonoid C-glycosides, and/or flavonoid O-, C-glycosides (*Niessen, 2006*). A total of seven different flavonols were identified and characterized from red onions; however, concentrations of flavonoids vary among genotypes and species *(Bonaccorsi et al., 2005)*. These flavonoids determine the color of the onion bulb and tolerance to biotic and abiotic stresses in alliums (Khandagale & Gawande, 2019). The metabolomic approach in *Allium* is focused toward exploring compositional difference, characterization of enzyme activity, and detection and chemical assignment of metabolites in different genotypes and cultivars *(Schmidt, Nyberg & Staerk, 2014; Yoshimoto et al., 2015; Nakabayashi et al., 2016; Molina-Calle et al., 2017)*. Little work has been performed to explore the metabolic response of *Allium* with respect to abiotic and biotic stresses, which affect plant growth and yield. Recently, Abdelrahman et al. (2016) attempted the dissection of *Trichoderma l ongibrachiatum*-induced defense response of onion against *Fusarium through* metabolite profiling. More than 25 metabolites were accumulated in a significant amount in the plant when onion seeds were primed with *T. longibrachiatum*. These metabolites are produced in abiotic and biotic stresses and increase onion resistance to *F. oxysporum*. Therefore, there is a wide scope to explore these areas, which might help to decipher resistance and/or tolerance mechanisms in alliums.

### Microbial metagenomics

A large number of microorganisms inhabit around, on, or in plants in a natural ecosystem. The interaction of the microbial community with plants leads to plant growth promotion in different ways, such as nutrient mobilization and secretion of plant growth hormone, enzymes, antibiotics, and other beneficial compounds *(Verma et al., 2018)*. The exploration of microbial diversity interacting with crop-specific plants is limited because only a few percentage of microbes can be cultured and characterized; to explore a large number of microbes, the metagenomics approach can be applied as it is independent of culture *(Krishna et al., 2019)*. Metagenomics approaches are being applied to explore the significance of uncultivated microbes interacting with different *Allium* species *(Yurgel et al., 2018; Knerr et al., 2018; Qiu et al., 2018; Huang, 2018; Matthews, Pierce & Raymond, 2019)*. A recent study conducted by Chen et al. (2018) revealed that the crop rotation of Chinese cabbage (*Brassica rapa subsp. pekinensis*) with potato onion (*A. cepa var. aggregatum Don*.) significantly reduces the incidence and disease index of clubroot disease caused by *Plasmodiophora (Plasmodiophora brassicae*) in Chinese cabbage. A reduction in disease was observed due to the reduction in secondary plasmodia of *Plasmodiophora*. A similar study conducted by Nishioka et al. (2019) revealed that cucumber crop rotation and mixed cropping with *Allium* species (*A. cepa* and *A. fistulosum*) suppress the *Fusarium* wilt. 16S rRNA gene sequencing by using Illumina MiSeq revealed the dominant presence of the *Flavobacterium* genus. The predominance of *Flavobacterium* inhibits the multiplication of pathogens in soil (Nishioka et al., 2019). The metagenomics study of Chinese leek (*A. tuberosum*) by using high-throughput 16S rRNA gene Illumina sequencing revealed the predominance of *Proteobacteria, Acidobacteria, Bacteroidetes, Cyanobacteria ,* and *Planctomycetes* microbial communities responsible for potential antifungal and nematicidal activities (Huang, 2018). The metagenomics of onion bulb was conducted to study bulb-rotting bacterial and fungal pathogens during storage; as under storage environment, a significant amount of onion bulbs are affected by diseases. The metagenomics for potential pathogen analysis, which was performed for both diseased and healthy bulbs under storage conditions, revealed the abundance of the following bacterial and fungal taxa: *Acinetobacter, Burkholderia, Citrobacter, Enterobacteriaceae, Gluconobacter, Pseudomonas, Botrytis, Nectriaceae, Penicillium, Wickerhamomyces* and *Candida*. These taxa are also abundant in the disease-free bulb, but the presence of various fermenters in diseased bulbs suggests that fermenters play a crucial role in onion bulb rotting, along with abiotic factors *(Yurgel et al., 2018)*. These studies have highlighted the significance of alliums metagenomics; in the future, alliums metagenomics should be further explored to identify bacterial and fungal strains exhibiting bactericidal, fungicidal, nematicidal, and growth-promoting activities as well asgenes and proteins for crop protection and production. Till date, the rhizosphere of *Allium* crops is largely unexplored, and the metagenomics approach can help in discovering the diversity as well as an abundance of microbes associated with *Allium* roots in varying soil type and climatic conditions.

### Meta-omics approach

**Figure 3:**
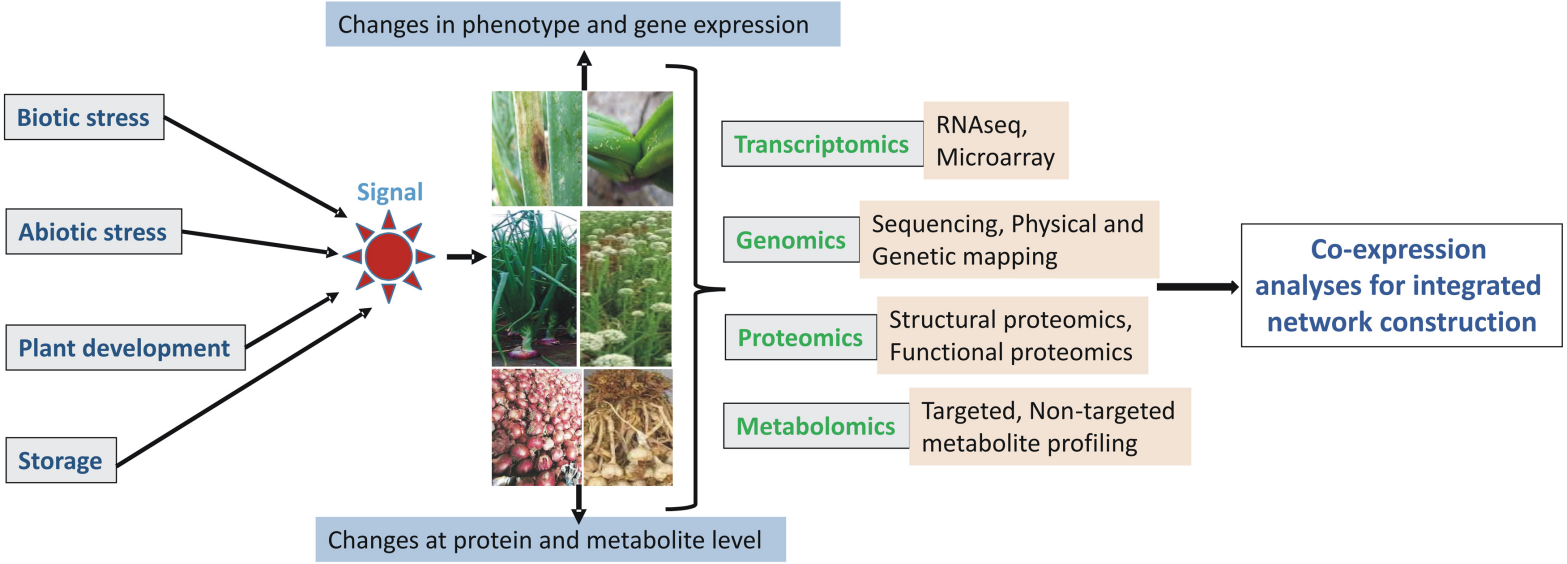
Schematic representation of integrated ‘omics’ analyses in *Allium* crops.

The information generated from these individual omics experiments needs to be correlated with each other to draw meaningful and precise conclusions for further knowledge-based breeding for elite crop development. The omics era has revolutionized research in complex biological processes and traits in many model organisms. Thus, we need to perform integrated omics studies in *Allium* crops for a quick and improved understanding of molecular mechanisms of various traits (Fig. 3). Integrated transcriptome and proteome have been performed in garlic for studying flower and pollen development *(Shemesh-Mayer et al., 2015)*.

Similarly, RNA-seq and metabolomics have been integrated into the study of the abiotic stress response *(Abdelrahman et al., 2015)*, onion bulb color (Zhang et al., 2018), and flavonoids *(Abdelrahman et al., 2019)*. From these studies, authors have suggested that separate omics analyses by using any single approach can be insufficient for the precise elucidation of various traits and their molecular mechanisms. These integrated omics interventions further enable researchers to precisely associate genetic variation with a metabolite or protein and thus ultimately help in selecting candidate genes with greater confidence for future metabolic and protein engineering in *Allium* crops for the development of noble varieties.

### Conclusion and future perspective

Although *Allium* crops are widely cultivated and consumed, research in these crops is challenging due to their biennial nature, cross-pollination, inbreeding depression, photoperiod sensitivity, and lack of flowering. However, with the development of advanced omics technologies and their affordability, research in these crops is gaining attention and pace by overcoming these challenges.

Due to the large size of the genome in *Allium* species, the availability of genome sequences can be a major breakthrough in *Allium* research. Rapid progress in sequencing technologies can definitely enable researchers to complete *Allium* sequencing projects. Increased number of doubled haploid lines need to be developed for alliums growing in different photoperiod conditions and used for genome sequencing. Genome sequencing projects for onion and shallots are under progress at Wageningen University and Yamaguchi University, respectively. To complete these daunting tasks, a coordinated effort is needed. The plastid genome of several *Allium* species has been characterized and needs to be used for male sterility and phylogenetic analyses. With the advancement in sequencing technologies and reduction in cost, recently, the use of *RNA-seq* has significantly increased in diverse alliums*.* These studies have attempted the elucidation of complex molecular mechanisms involved in male sterility, bulb development, bulb color, flowering, and stress responses in alliums*.* Several putative unigenes conferring the aforementioned traits have been identified from *RNA-seq* datasets. Biotic and abiotic stresses are the key factors that affect both the quality and yield of *Allium* crops; yet, limited information is available regarding molecular response to stress in these crops. Thus, transcriptomic analyses under stress will help in understanding the stress physiology and molecular biology of alliums. miRNAs play a crucial role in gene regulation in plant development and stress response in plants; however, information on small RNAs is also lacking in alliums. Generally, wild relatives harbor several economically vital traits, such as abiotic stress tolerance, disease resistance, and secondary metabolites. Thus, wild alliums need to be investigated using transcriptomics to identify genes responsible for these traits as well as those for domestication. Further, these domestication-associated genes can be modified using transgenic or genome editing tools to cultivate these wild relatives. The level of proteins and metabolites often differ with the mRNA expression profile of corresponding genes. Therefore, proteomic and metabolomic analyses are crucial because they analyze the end product of the central dogma, and thus, these omics tools should be used in an integrated manner for the proper unraveling of the trait. A few studies have reported the use of proteomics and metabolomics in understanding biological processes, such as stress response, flowering, and metabolic profiling for geographic origin authentication and analyses of OSCs. However, these efforts are not sufficient, and a few areas, such as bolting in onion; flowering in short-day garlic; storability of onion and garlic; high TSS onion for processing, pungency; and aroma, disease and pest resistance need to be addressed using these omics technologies. These studies need to be conducted comparatively on short-day and long-day onions to obtain some clues on the domestication of onion and garlic. Recently developed breeding techniques, such as CRISPR, may be used in alliums for targeted genome editing of key genes involved in haploid induction, male sterility, and virus resistance. However, to validate specificity of mutations, a reference genome is required; thus,the research fraternity working on *Allium* crops is eagerly waiting for the release of the draft sequence of onion. Availability of sequence information can open new avenues and accelerate *Allium* research, such as molecular breeding, functional genomics, and gene editing.

##  Supplemental Information

10.7717/peerj.9824/supp-1 Supplemental Information 1 Onion transcription factor analysis

10.7717/peerj.9824/supp-2 Supplemental Information 2 Metabolites identified in Alliums, their applications and functions

## Additional Information and Declarations

### Data Availability

The following information was supplied regarding data availability: This is a literature review and thus does not have any raw data.
