# Supplementary material for: Omics approaches in Allium research: Progress and way ahead
Source: PeerJ. 2020 Sep 9;8:e9824. doi: 10.7717/peerj.9824 (PMC7486827; doi:10.7717/peerj.9824)
Supplement: Supplemental Information 2 [file peerj-08-9824-s002.docx]

**Supplementary table 1: Metabolites identified in Alliums, their applications and functions**

| **Secondary metabolite identified** | **Application/Function** | **Purpose of study** | **Reference** |
| --- | --- | --- | --- |
| Alliin, | Antineoplastic and Hypoglycemic Agent | Geographical classification of garlic for authentic identification | *Hrbek et al 2019* |
| phosphatidylcholine | metabolism and signalling |  |  |
| arginine | essential amino acid |  |  |
| Dehydroalanine | uncommon amino acid found in peptides of microbial origin |  |  |
| L-γ-Glutamyl-S-allyl-L-cysteine | It precursors for allium flavour |  |  |
| choline glycerolphosphate | Major forms of choline storage |  |  |
| 1-propenyl methyl disulphide | Constituent of the essential oils of Allium species. | Pickling of garlic has changed metabolome than fresh garlic | *Farag et al 2019* |
| Dimethyl trisulfide | volatiles emitted from cooked  Alliums |  |  |
| 2-Acetylpyrrole | contributes to many aroma |  |  |
| Methyl pentyl disulfide | Flavouring Agent in spices |  |  |
| Allyl methyl trisulfide | constituent of Allium species |  |  |
| Dimethyl tetrasulfide | Flavouring Agent in spices |  |  |
| 3,5-Diethyl-1,2,4-trithiolan | Flavoring Agents |  |  |
| Diallyl sulphide | Flavouring ingredient. Neutriceutical with anti-cancer properties. | Antimicrobial activities and profiling of phytochemicals | *Farag et al 2017* |
| p-Cuminaldehyde | It has a role as an insecticide, a volatile oil component and a plant metabolite |  |  |
| 3-Vinyl-1,2-dithiacyclohex-5-ene | Transformation product of allicin |  |  |
| Kaempferol | Antioxidant by reducing oxidative stress, anticancer |  |  |
| Isorhamnetin | methylated metabolite of quercetin |  |  |
| Quercetin | antioxidant and anti-inflammatory effects |  |  |
| Allithiamine | lipid-soluble form of vitamin B1 |  |  |
| Diethylphthalate | Used in cosmetics, insecticides, and aspirin. |  |  |
| Alanine | Alanine is a non-essential amino acid | Compositional differences between fresh and black garlic | *Molina-Calle et al 2017* |
| Muramic acid | characteristic polysaccharide composing bacterial cell wall |  |  |
| Norharman | It has a role as a marine metabolite and a fungal metabolite. |  |  |
| Acetopine | Found in fats and oils. |  |  |
| Allicin | Antibacterial and anti-fungal properties. Allicin is garlic's defence mechanism against attacks by pests. |  |  |
| Betaine | modified amino acid, has role in abiotic stress |  |  |
| Guanosine | Involved in signal transduction pathway |  |  |

(Functions were taken from <https://pubchem.ncbi.nlm.nih.gov/>)
